# A General One-Pot Approach to Synthesize Binary and Ternary Metal Sulfide Nanocrystals

**DOI:** 10.1186/s11671-019-2856-7

**Published:** 2019-01-11

**Authors:** Chao Xiong, Mingrui Liu, Xifang Zhu, Aiwei Tang

**Affiliations:** 1 School of Electrical and Photoelectronic Engineering, Changzhou Institute of Technology, Changzhou, 213032 China; 2 Key Laboratory of Luminescence and Optical Information, Ministry of Education, School of Science, Beijing Jiao Tong University, Beijing, 100044 China

**Keywords:** One-pot approach, Metal sulfide, Nanocrystals, Metal-thiolate, Photoluminescence, Synthesis

## Abstract

A general one-pot approach is developed to synthesize a series of binary metal sulfide nanocrystals (NCs) including PbS, Cu_2_S, ZnS, CdS, Ag_2_S, and ternary CuInS_2_ and CdS:Cu(I) NCs. This synthetic approach involves thermal decomposition of the mixture of inorganic metal salts and *n*-dodecanethiol (DDT) without pre-synthesis of any organometallic precursors. In this method, layered metal-thiolate compound is formed at the beginning of the reaction and then this intermediate compound is decomposed into small particles, leading to further growth as the reaction time increases. The as-obtained CdS NCs exhibits a broad but weak surface-state emission, and the Cu(I) doping leads to a red-shift of the emission band due to the Cu(I)-related emission. It is expected that this one-pot approach can be extended to prepare multinary metal sulfide NCs.

**Electronic supplementary material:**

The online version of this article (10.1186/s11671-019-2856-7) contains supplementary material, which is available to authorized users.

## Background

In the past few decades, colloidal inorganic NCs have attracted much attention due to their unique optical and electrical properties [1, 2], as well as their wide potential applications in light-emitting diodes [3–6], biological labels [7–9], solar cells [10–13], memory devices [14–16], and so on*.* As an important category of colloidal inorganic NCs, metal sulfide have exhibited distinct size-, structure-, and composition-dependent optoelectronic properties [2]. Therefore, it is necessary to develop a general and simple synthetic approach that yields monodispersed metal sulfide, in which the size, shape, phase, and chemical composition can be controlled precisely by changing the starting materials and reaction conditions. Up to date, a variety of synthetic methods, including hydrothermal or solvothermal techniques [17, 18], hot-injection approaches [19, 20], and single-source precursor routes [21, 22], have been reported to prepare different types of high-quality colloidal inorganic NCs. However, it is difficult to control the shape and size in the hydrothermal or solvothermal methods, and the air-free manipulation and fast injection rate limits the large-scale production in the hot-injection routes. In addition, the precursors should be pre-synthesized in the thermal decomposition of single-source methods, which will add some additional steps during the synthesis [23]. Therefore, it is still challenging to develop a more simple, low-cost, and general wet-chemical synthetic route for preparing different types of inorganic NCs. Li’s group developed a facile “dispersion-decomposition” route to synthesize high-quality metal sulfides using inorganic salts and alkylthiol as the raw materials [24]. However, these approaches mainly focused on synthesis of binary metal sulfide NCs, and the synthesis of doped and ternary metal sulfide NCs by a simple and versatile approach is less studied. Moreover, the formation of gelification phenomena is also less discussed. Recently, our group developed a simple and versatile method for preparing a wide range of metal sulfide NCs and some heterostructured NCs [25–28]. To demonstrate the universality of this one-pot method, herein, this one-pot approach is developed to synthesize a series of binary metal sulfide NCs including PbS, Cu_2_S, ZnS, CdS, Ag_2_S and ternary CuInS_2_ NCs, and doped CdS:Cu(I) NCs. This process does not need any extra steps for pre-synthesis of toxic organometallic precursors, and DDT is chosen not only as sulfur source but also as a surface-capping agent without any phosphine agents. A layered metal-thiolate compound is produced upon heating the inorganic salt and DDT, which then is decomposed into nanocrystal nuclei. The metal-thiolate compounds are air-stable and become a gel at room temperature. The nucleation and growth process can be tuned by changing the reaction conditions, resulting in controllable shape, size, and chemical composition.

## Methods

### Synthesis of Binary Metal Sulfide NCs

For a typical synthesis of PbS nanocrystals, 3 mmol of Pb(OAc)_2_·3H_2_O and 20 mL of DDT were added into a three-necked flask at room temperature, and then the mixture was degassed by nitrogen gas following about 20 min. Afterwards, the mixture was heated to 200 °C and maintained for 60 min. After reaction, it was terminated by naturally cooling down to room temperature after removal of the heating equipment. The nanocrystals can be separated after adding some ethanol by centrifugation at 7000 rpm for 10 min. The precipitates were washed using chloroform to remove precursor and surfactant residuals. The above centrifugation and purification procedures were repeated twice, and then the samples were re-dispersed into chloroform or dried in vacuum for subsequent characterization.

For synthesis of Cu_2_S nanocrystals, 3 mmol of Cu(acac)_2_ was added into 10 mL of DDT and 20 mL of ODE in a three-necked flask, and then the mixture was heated to 200 °C and kept for 60 min.

For synthesis of ZnS nanocrystals, 3 mmol of Zn(acac)_2_ was added into 5 mL of DDT and 25 mL of ODE in a three-necked flask, and then the mixture was heated to 240 °C and kept for 180 min.

For synthesis of CdS nanocrystals, 5 mmol of Cd(acac)_2_ and 30 mL of DDT were added into a three-necked flask, and then the mixture was heated to 200 °C and kept for 23 h.

For synthesis of Ag_2_S nanocrystals, 3 mmol of Ag(OAc) and 20 mL of DDT were added into a three-necked flask at room temperature, and then the mixture was heated to 205 °C and kept for 100 min.

### Synthesis of Ternary Metal Sulfide NCs

For synthesis of CdS:Cu(I) nanocrystals, 4.5 mmol of Cd(acac)_2_ and 0.5 mmol Cu(acac)_2_ were added into 30 mL of DDT in a three-necked flask, and then the mixture was heated to 200 °C and kept for 23 h.

For synthesis of CuInS_2_ nanocrystals, 3.1 mmol of Cu(acac)_2_, 1.9 mmol of In(acac)_3_, 5 mL of DDT and 25 mL of ODE were added into a three-necked flask, and then the mixture was heated to 240 °C and kept for 60 min.

All the detailed experimental conditions for different products in our work are summarized in Table 1.

**Table 1:** Detailed experimental conditions for synthesis of different types of metal sulfide NCs

Samples	Metal precursors	DDT	ODE	Temperature	Morphology
PbS	3 mmol Pb(OAc)_2_ xH_2_O	20 mL	–	200 °C	Octahedral
Cu_2_S	3 mmol Cu(acac)_2_	10 mL	20 mL	200 °C	Spherical
ZnS	3 mmol Zn(acac)_2_	5 mL	25 mL	240 °C	Shapeless
CdS	5 mmol Cd(acac)_2_	30 mL	–	200 °C	Near-spherical
Ag_2_S	3 mmol Ag(OAc)	20 mL	–	205 °C	Spherical
CdS:Cu(I)	4.5 mmol Cd(acac)_2_ + 0.5 mmol Cu(acac)_2_	30 mL	–	200 °C	Near-spherical
CuInS_2_	3.1 mmol Cu(acac)_2_ + 1.9 mmol In(acac)_3_	5 mL	25 mL	240 °C	Bullet

### Characterization

The size and shape of the samples were examined by using a transmission electron microscope (TEM; Hitachi-7650) with the accelerating voltage of 100 kV and high-resolution transmission electron microscope (HRTEM; JEM-2010) operating at an accelerating voltage of 200 kV. The crystalline structure of the as-obtained products was determined by using a Bruker D8 Advance X-ray Diffractometer (XRD) with Cu Kα radiation (λ = 1.54056 Å). The chemical composition and valance-state of the samples were measured using a VG Scientific ESCALab220i-XL X-ray photoelectron spectrometer (XPS) with a 300 W Al Kα radiation source. All binding energies for different elements were calibrated with respect to C1s line at 284.8 eV from the contaminant carbon. The UV-Vis absorption spectra measurements of the samples in chloroform solution were carried out using a Shimadzu-UV 3101 spectrophotometer and the fluorescent spectra were recorded using a Varian Cary Eclipse fluorescence spectrophotometer.

## Results and Discussion

**Fig. 1:**
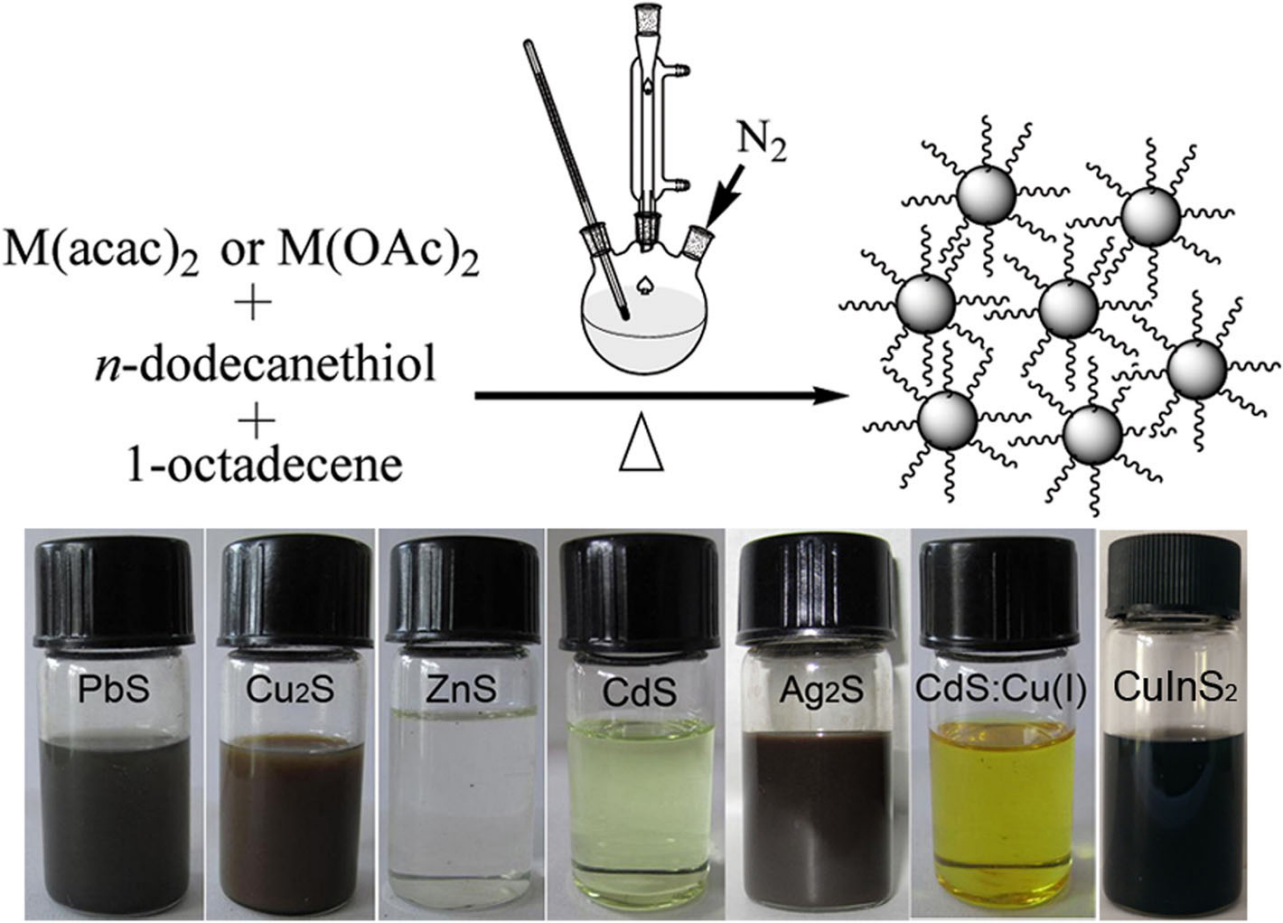
Top panel, schematic illustration of the synthetic procedure for different inorganic NCs; bottom panel, photographs of different products dispersed in chloroform solutions at room temperature

**Fig. 2:**
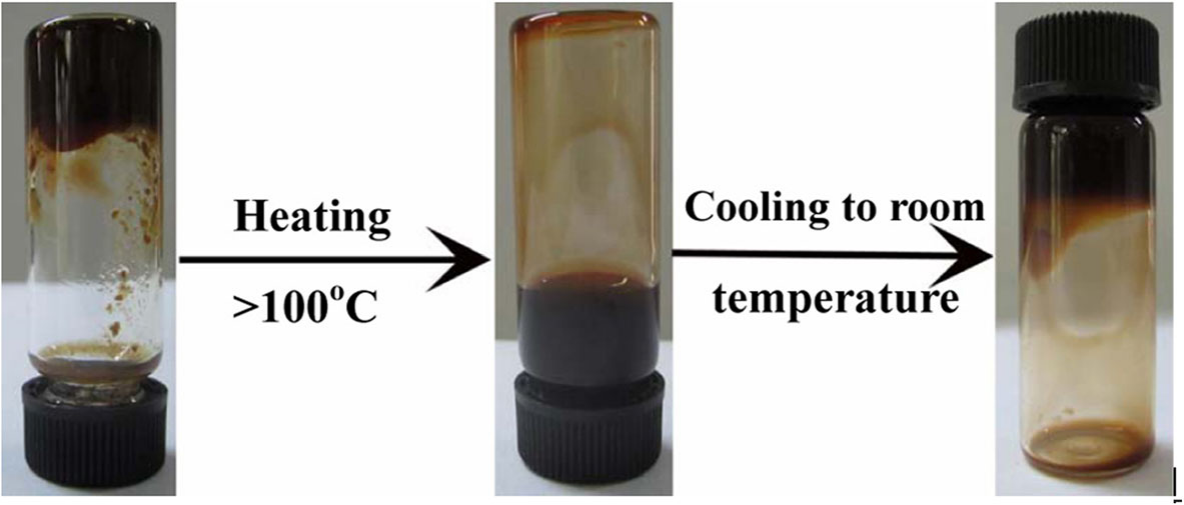
Photographs of the state change of Cu-thiolate compound obtained at the initial stage of the reaction

A schematic general synthetic procedure of different metal sulfide NCs is illustrated in the top panel of Fig. 1. The bottom panel of Fig. 1 shows the digital photographs of chloroform solutions of different products synthesized in our work. These samples can be well dispersed in chloroform to form homogenous colloidal solutions and exhibit different colors at room temperature. In this one-pot reaction, DDT acted as not only the sulfur source, but also the capping-agent and reaction media, even it was used as the reducing agent in the synthesis of binary and ternary metal sulfide NCs. ODE was used as a reaction media to allow the reaction to be performed. Generally, gelification phenomena were observed from the aliquots extracted at the initial reaction stages after they were cooled down to room temperature. When the gel was heated to above 100 °C, the gel became fluid. Unexpectedly, the aliquots became gels again at room temperature. We take Cu_2_S NCs as a typical example, the digital pictures of the states of matter for aliquot extracted at 180 °C are given in Fig. 2. As stated in previous reports that an intermediate compound was formed at a relatively low reaction temperature during this reaction, and then decomposed into nuclei to promote the growth of the NCs [23]. In the synthesis of Cu_2_S NCs, the colors of the aliquots changed from yellow to dark-brown as the reaction proceeded, and the gelification phenomena disappeared after reaction for 10 min at 200 °C, which indicates that the formation of the gels is closely related with the intermediate compounds.

**Fig. 3:**
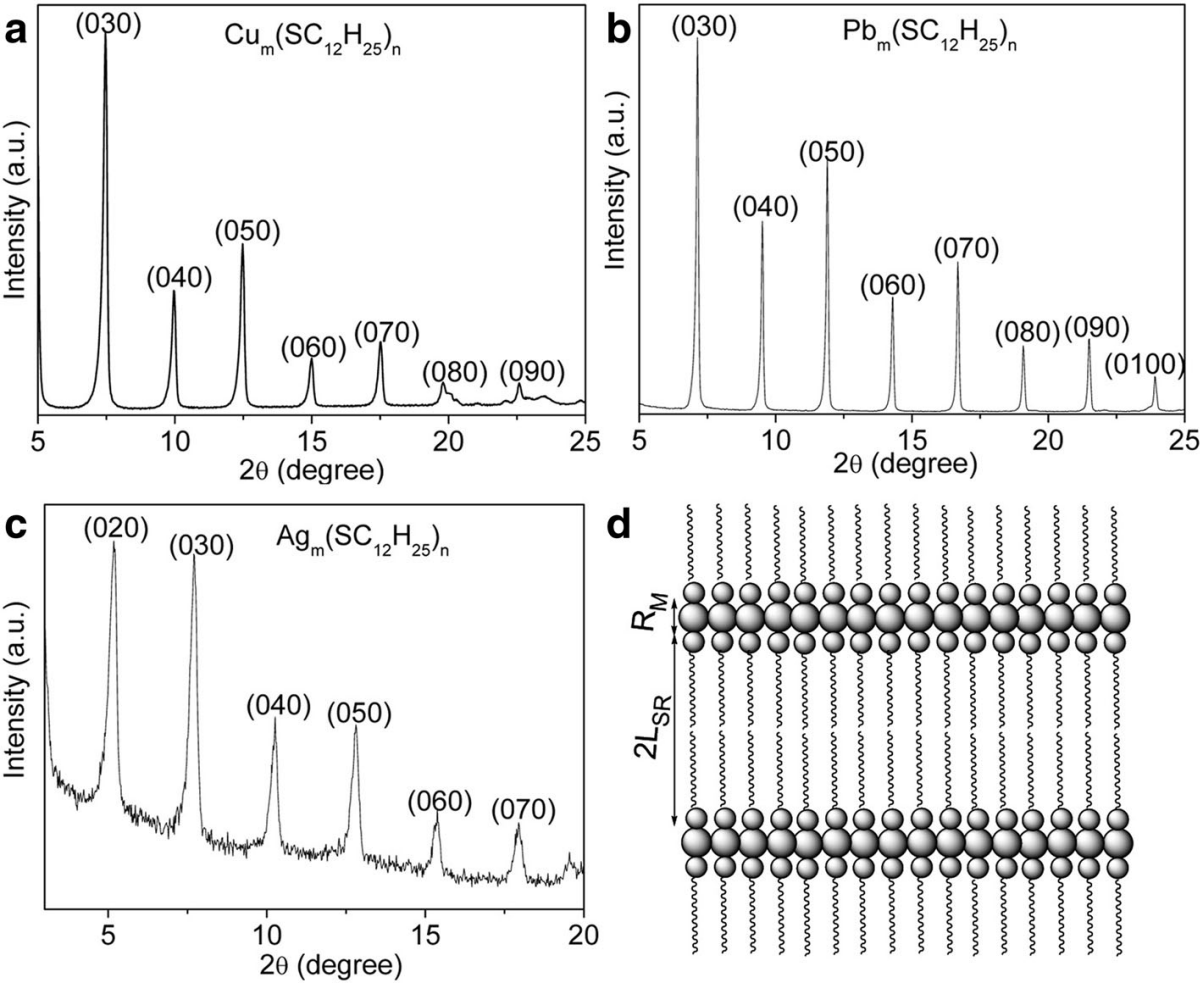
Representative XRD patterns of the intermediate compounds obtained at the early stage. **a** Cu-thiolate compound. **b** Pb-thiolate compound. **c** Ag-thiolate compound. **d** Scheme of the layered structure formed at the initial reaction stage

To further study the formation mechanism and structure of the intermediate compounds, some aliquots of the representative samples were extracted from the initial stage, and the corresponding XRD patterns are shown in Fig. 3a–c, respectively. A series of narrow and sharp diffraction peaks are clearly observed, which can be ascribed to the successive (*0 k0*) orders of reflections from a layered structure. According to Bragg’s law, the mean interlayer spacing between the two sharp diffraction peaks for the three representative samples is calculated to be about double-layer of DDT molecules and one layer of metal ions. The schematic illustration of the stacked structure for the metal-thiolate compound is depicted in Fig. 2d. Some small discrepancy between the calculated values and theoretical values for the three samples may result from the difference of diameters of metal ions and no interpenetration at the interface between layers. The aforementioned results indicate that the intermediate compounds formed at initial stage are layered metal-thiolate compound with polymeric structures, which can be demonstrated by the gelification phenomena [23].

**Fig. 4:**
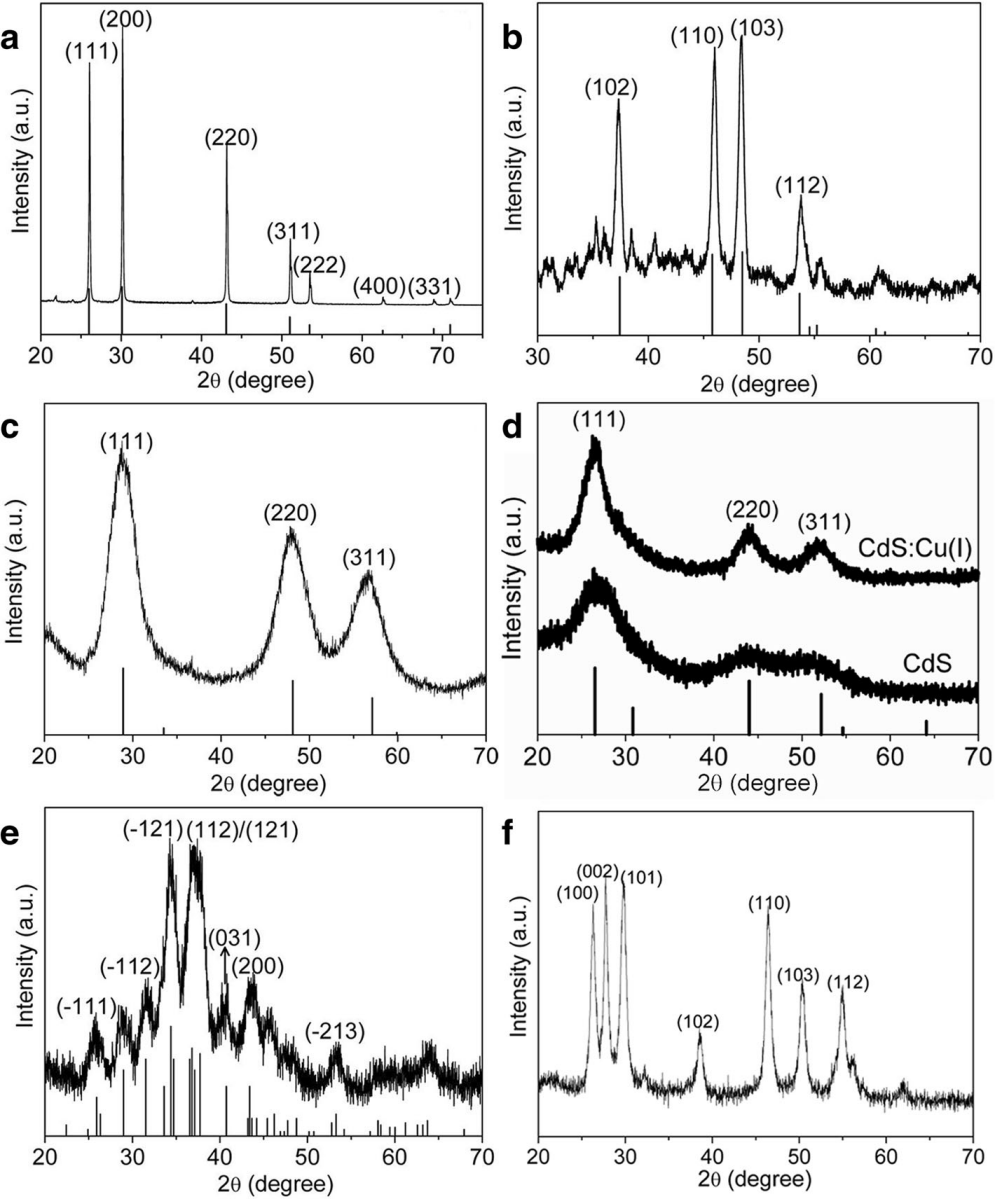
XRD patterns of the as-obtained different metal sulfide NCs and the corresponding standard diffraction lines are placed at the bottom. **a** PbS. **b** Cu_2_S. **c** ZnS. **d** CdS and CdS:Cu(I). **e** Ag_2_S. **f** CuInS_2_

The crystal structures of the as-obtained products were confirmed by the XRD patterns. Figure 4 shows the XRD patterns of PbS, Cu_2_S, ZnS, CdS:Cu(I), Ag_2_S, and CuInS_2_ NCs. As shown in Fig. 4a, the as-observed diffraction peaks can be assigned the face-centered-cubic (fcc) structure of PbS (JCPDS 77-0422), and no other phases are present in the patterns. The XRD pattern of Cu_2_S shown in Fig. 4b is consistent with the standard pattern of hexagonal Cu_2_S (JCPDS no. 26-1116). For ZnS NCs (shown in Fig. 4c), the three major diffraction peaks confirm the cubic zinc blende structure according to the standard pattern of the bulk ZnS (JCPDS 80-0020). The broadening of the XRD peaks indicates the nature of small size. Similarly, Fig. 4d depicts the XRD patterns of the CdS and CdS:Cu(I) NCs, and their positions and relative diffraction intensities match well with those from the standard patterns of CdS (JCPDS 10-0454), demonstrating that the CdS:Cu(I) NCs possess a zinc blende structure similar to CdS NCs. There is little difference observed in the XRD patterns of CdS and CdS:Cu(I) NCs, indicating the incorporation of Cu(I) ions into CdS lattice has little influence on the crystal structure. Figure 4e depicts the XRD pattern of Ag_2_S NCs, all the diffraction peaks are consistent with the standard pattern of monoclinic Ag_2_S (JCPDS card no. 14-0072), which indicates that the as-obtained samples are in pure Ag_2_S phase. It is well known that the Ag NCs can be obtained in the presence of DDT due to the reducing ability; however, the nucleophilic attack of DDT also contributes to the formation of Ag_2_S NCs, which was studied comprehensively in our previous work. In the case of this work, pure Ag_2_S NCs could be obtained by directly heating the Ag(OAc) in the pure DDT without any surfactant at 200 °C. For the CuInS_2_ NCs shown in Fig. 4f, all the diffraction peaks match well with the wurtzite phase. As a matter of fact, the crystal structure of the ternary CuInS_2_ NCs could be tuned by varying the In sources and the Cu/In precursor ratios [27].

**Fig. 5:**
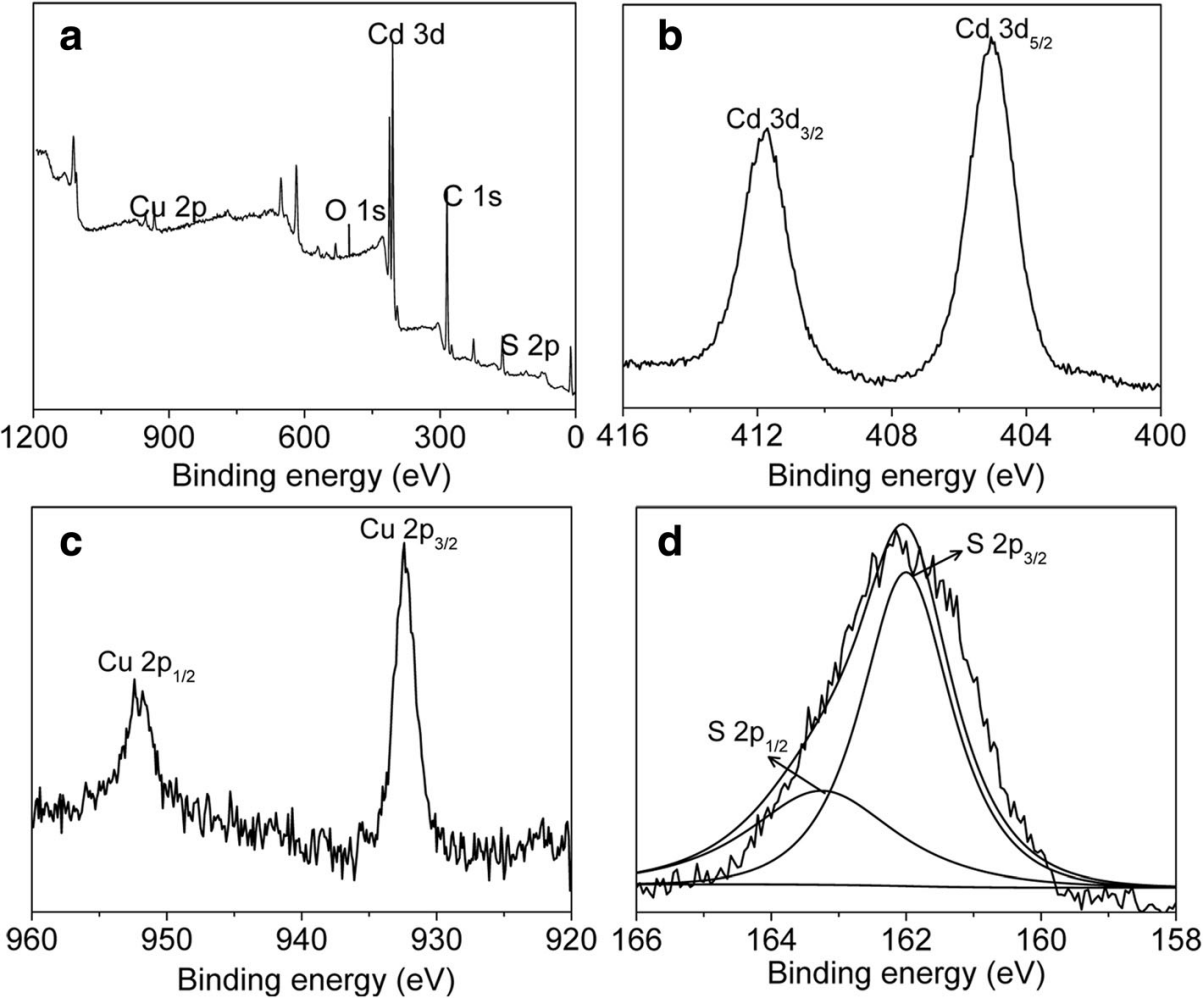
XPS spectra of CdS:Cu(I) NCs. **a** Survey spectrum. **b** Cd 3d. **c** Cu 2p. **d** S 2p

Due to the complexity of the valence states of Cu ions, it is worthy to figure out the valence state of Cu ion in CdS:Cu samples. XPS spectra were used to characterize the chemical composition and the valence state of Cu ions in the samples, and Fig. 5 shows the XPS results of CdS:Cu NCs. The survey XPS spectrum of the samples shown in Fig. 5a demonstrates the presence of Cd, S, and Cu components in the as-obtained samples. By analyzing the XPS signal of Cu 2p shown in Fig. 5c, it is found that two peaks are located at 952 eV and 932.4 eV, corresponding to Cu 2p_1/2_ and Cu 2p_3/2_ signals, respectively. This result suggests the presence of Cu ion in + 1 state according to previous reports [23]. As the “shake-up” peak is absent between the Cu 2p_3/2_ and Cu 2p_1/2_ signals at around 942 eV, the possibility of + 2 state for Cu ion can be ruled out [29]. Therefore, the analysis mentioned above supports that Cu ion exists in + 1 state in CdS:Cu(I) NCs.

**Fig. 6:**
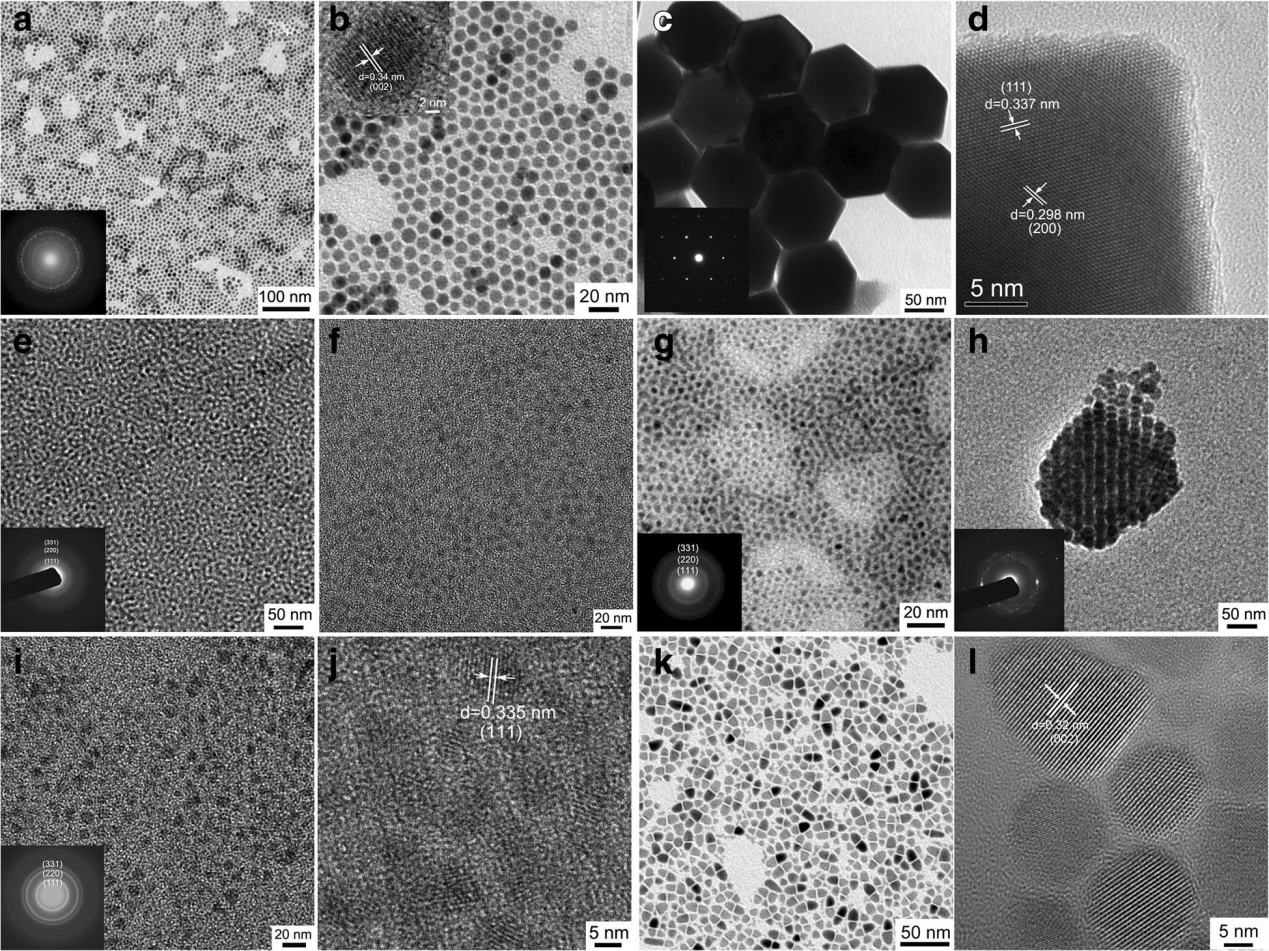
TEM images of the as-collected different products. **a**, **b** Cu_2_S. **c**, **d** PbS. **e**, **f** CdS. **g** ZnS. **h** Ag_2_S. **i**, **j** CdS:Cu(I). **k**, **l** CuInS_2_. The insets of **a**, **c**, **e**, **g**, **h**, and **i** are the corresponding SAED patterns, and the inset of **b** is the corresponding HRTEM image

TEM technique was employed to ascertain the morphology and size of the as-obtained NCs. Figure 6 shows the TEM images of Cu_2_S, PbS, CdS, ZnS, Ag_2_S, CdS:Cu(I), and CuInS_2_ NCs. As shown in Fig. 6a, b, the as-obtained Cu_2_S NCs exhibit a spherical shape with a mean diameter of 8.0 nm and the size distribution is less than 7%, and the samples reveal a self-assembly behavior of hexagonal close-packed array. The selected-area electron diffraction (SAED) pattern depicted in the inset of Fig. 6a exhibits polycrystalline diffraction rings that can be indexed to hexagonal Cu_2_S (JCPDS no. 26-1116), which is in good agreement with the XRD result. Clear lattice fringes visible in the HRTEM image (inset of Fig. 6b) confirm their good crystallinity, and the interplanar spacing of 0.34 nm corresponds to (002) planes of a hexagonal Cu_2_S phase. Figure 6c, d displays the TEM images of PbS NCs. At first glance, the NCs are hexagonal in shape (shown in Fig. 6c). As a matter of fact, however, they are hexagonal projections of octahedrons with a mean diameter of 93.6 nm. The corresponding SAED pattern depicted in the inset of Fig. 6c demonstrates the nature of single crystals. For a typical HRTEM image of octahedral PbS NCs, we can observe obvious lattice fringes with interplanar spacings of 0.337 and 0.298 nm, which are ascribed to (111) and (200) planes of a fcc PbS phase, respectively. Figure 6e–g are the TEM images of CdS and ZnS NCs, and the samples are quasi-spherical shape with the average size less than 5 nm. The corresponding SAED patterns confirm their cubic zinc blende structure. The TEM image of Ag_2_S NCs is shown in Fig. 6h, and the NCs are spherical in shape with a mean diameter of about 7 nm. The SAED shown in the inset of Fig. 6h indicates polycrystalline diffraction rings that can be indexed to the monoclinic structure of Ag_2_S, which is in consistent with the XRD result. Figure 6i depicts the TEM image of CdS:Cu(I) NCs, and the shape is quasi-spherical and the mean size is less than 5 nm, and the SAED shown in the inset can be indexed to pure cubic phase structure. The HRTEM image (in Fig. 6j) of CdS:Cu(I) NCs shows the resolved lattice fringe with the interplanar spacing of 0.335 nm assigned to the (111) plane of cubic structured CdS. The great difference in size and shape of these metal sulfide nanocrystals may arise from the different decomposition rates of metal-thiolate compounds. Figure 6k presents the low-magnified TEM images of ternary CuInS_2_ NCs, and all the samples exhibit a bullet shape. The corresponding HRTEM image shown in Fig. 6l indicates that the interplanar distance is about 0.32 nm, which accords with the plane distance of (002) in wurtzite CuInS_2_ phase.

**Fig. 7:**
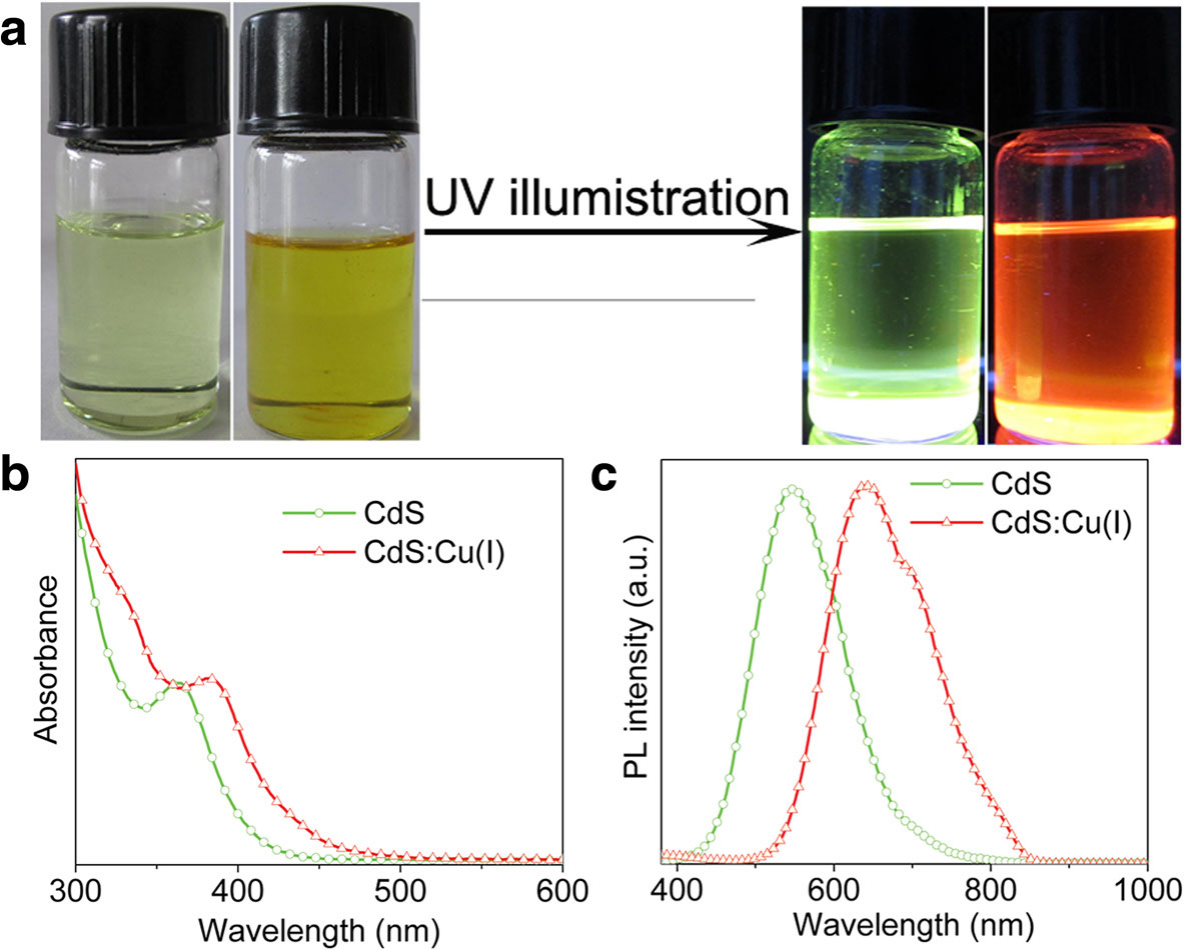
**a** Digital pictures of CdS and CdS:Cu(I) nanocrystals solution in chloroform before (left) and after (right) irradiation under a 365 nm UV lamp. **b** UV-Vis. **c** Fluorescence emission spectra of CdS and CdS:Cu(I) NCs, and the excitation wavelength is 350 nm

**Fig. 8:**
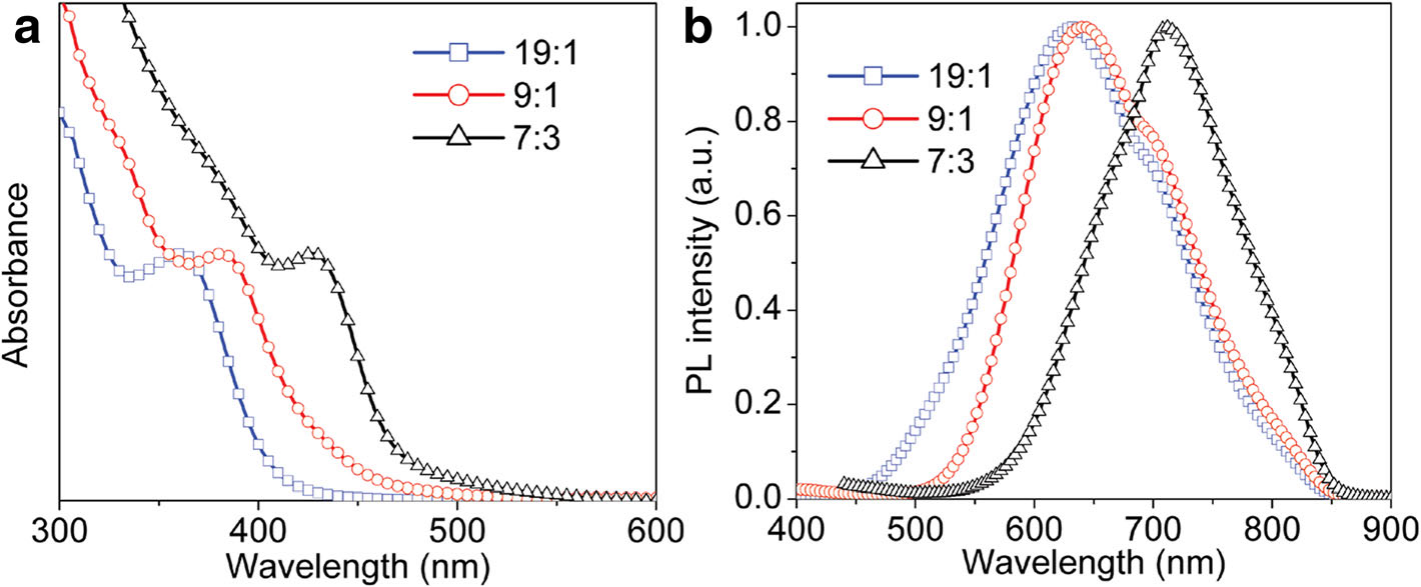
**a** Absorption. **b** PL spectra of CdS:Cu(I) NCs with different Cu doping levels, and the excitation wavelength is 350 nm

UV-Vis absorption and fluorescence emission spectroscopy has often been employed to study quantum-confinement effect in semiconductor NCs. In addition, the fluorescence emission spectroscopy is also used to probe the defects or surface traps in the NCs. It is reported previously that some defects were introduced into the semiconductor NCs using DDT as sulfur source [30, 31]. Figure 7a shows the photographs of CdS and CdS:Cu(I) NCs before and after 365 nm UV irradiation. It is clearly observed that the colloidal solution of CdS NCs exhibits green emission under UV illumination, and the relative photoluminescence quantum yield (PLQY) is estimated to be about 10%. After doping Cu (I) ions into CdS NCs, the colloidal solution exhibits a relatively strong red emission, which demonstrates that the Cu (I) doping into CdS NCs can effectively tune the optical property of the semiconductor NCs. The UV-Vis absorption spectra of CdS and CdS:Cu(I) NCs are plotted in Fig. 7b. The absorption spectrum of CdS NCs shows a distinct absorption maximum at 364 nm, which is blue-shifted than bulk CdS (the band gap is 2.4 eV). In contrast, the absorption maximum of CdS:Cu(I) NCs shifts to 384 nm, exhibiting a clear red-shift as compared to that of pure CdS NCs. Due to the similar particle size of CdS and CdS:Cu(I) NCs from the TEM results, the red-shift of absorption maximum is not associated with the size of the NCs but is closely related with the doping Cu(I) ions into CdS NCs. The fluorescence emission spectra of CdS and CdS:Cu(I) NCs are given in Fig. 7c. The fluorescence emission spectrum of CdS NCs exhibits a broad band at 548 nm, which is attributed to the trapped surface state’s emission [32].The trapped surface state’s emission may originate from the localized surface states of the samples, which presumably are formed by the lack of bonding to S^2−^ due to the excess amount of DDT used in our experiment. For the fluorescence emission spectrum of CdS:Cu(I) NCs, a red-emissive maximum of 642 nm is observed, and the relative PLQY is estimated to be about 16%, which can be attributed to Cu (I)-related emission due to the recombination of an excited electron in the conduction band of the CdS NCs and a hole from the d-orbital of Cu ions [33]. The Cu(I) doping level plays an important role in the optical properties of CdS:Cu(I) NCs, and thus different CdS:Cu(I) NCs were synthesized by using different feeding ratios Cd/Cu precursors, such as 7:3, 9:1, and 19:1. The actual percentage of [Cu]/[Cu + Cd] can be estimated to be 12.3%, 6.8%, and 2.8% based on the XPS results shown in Additional file 1: Figure S1, which depicts the survey XPS spectra and Cu 2p signal of the CdS:Cu(I) NCs synthesized in the presence of different amount of Cu precursors. The corresponding absorption and PL spectra are given in Fig. 8a, b, and the absorption maximum shifts to longer wavelength with an increase of Cu(I) doping level (Fig. 8a). Moreover, such a red-shift is also observed in the PL maximum with an increase of Cu doping level from 2.8% to 12.3% (Fig. 8b). It should be noted that the PL emission band at 710 nm becomes dominant for the CdS:Cu(I) synthesized in the presence of Cd/Cu feeding ratio of 7:3, which indicates that the luminescence mainly derives from deep donor-acceptor recombination due to the incorporation of more Cu(I) ions into the CdS core.

## Conclusions

In summary, we reported a simple and general one-pot approach to synthesize binary and ternary metal sulfide NCs including Cu_2_S, PbS, ZnS, CdS, Ag_2_S, CdS:Cu(I), and CuInS_2_, in which no pre-synthesis organometallic precursors were required. In this reaction, layered metal-thiolate compounds were formed at the early stage, which effectively acted as the precursors to promote the growth of the NCs. The size and shape of the products could be controlled easily. Importantly, a high red-emissive CdS:Cu(I) was successfully synthesized through this one-pot route, which greatly reduced the operational complexity and offered an alternative method to prepare doped NCs. We believe that this versatile and simple one-pot strategy would open a new methodology for synthesis of other ternary or multinary metal sulfide NCs. More importantly, the different NCs synthesized using this approach exhibited different absorption regions and had distinct photoluminescence properties, making them good candidates for applications in photovoltaic devices and light-emitting devices.

## Additional file

Additional file 1: **Figure S1.** The XPS signals of (a) Full-scan and (b) Cu 2p for CdS:Cu NCs with different Cu doping levels. (DOCX 162 kb)

## Abbreviations

DDT: *n*-dodecanethiol
HRTEM: High-resolution transmission electron microscope
NCs: Nanocrystals
ODE: 1-octadecene
PL QY: Photoluminescence quantum yield
TEM: Transmission electron microscope
XPS: X-ray photoelectron spectrometer
XRD: X-ray diffractometer
