# Supplementary material for: A General One-Pot Approach to Synthesize Binary and Ternary Metal Sulfide Nanocrystals
Source: Nanoscale Res Lett. 2019 Jan 11;14:19. doi: 10.1186/s11671-019-2856-7 (PMC6329689; doi:10.1186/s11671-019-2856-7)
Supplement: Supplementary file 1 — Figure S1. The XPS signals of (a) Full-scan and (b) Cu 2p for CdS:Cu NCs with different Cu doping levels. (DOCX 162 kb) [file 11671_2019_2856_MOESM1_ESM.docx]

**Supporting Information**

**A General One-pot Approach to Synthesize Binary and Ternary Metal Sulfide Nanocrystals**

Chao Xiong^1^, Mingrui Liu^2^, Xifang Zhu^1^, Aiwei Tang^2^*

^1^ School of Electrical and Photoelectronic Engineering, Changzhou Institute of Technology, Changzhou, 213032, China

^2^Key Laboratory of Luminescence and Optical Information, Ministry of Education, School of Science, Beijing Jiao Tong University, Beijing 100044, China;

Email addresses for all authors:

Chao Xiong, Email: [xiongc@czu.cn](mailto:xiongc@czu.cn)

Mingrui Liu, Email: [17121678@bjtu.edu.cn](mailto:17121678@bjtu.edu.cn)

Xifang Zhu, Email: zhuxf@czu.cn

Aiwei Tang, Email: [awtang@bjtu.edu.cn](mailto:awtang@bjtu.edu.cn)

* Corresponding author (email: [awtang@bjtu.edu.cn](mailto:awtang@bjtu.edu.cn))

**Figure S1**

**
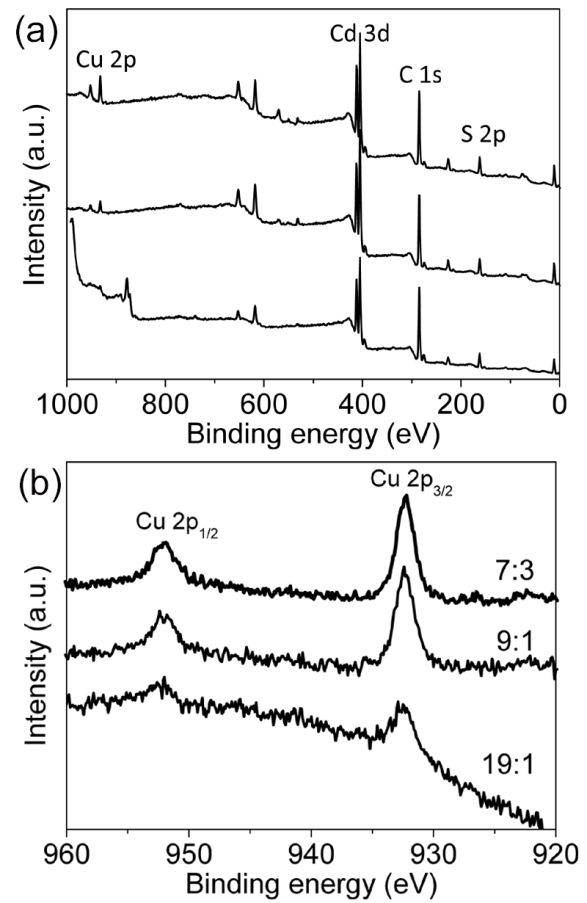
**

**Figure S1** The XPS signals of (a) Full-scan and (b) Cu 2p for CdS:Cu NCs with different Cu doping levels.
